# ASSOCIATION OF HEALTHCARE ACCESS AND DUAL ELIGIBILITY STATUS BASED ON ENROLLMENT IN MANAGED CARE

**DOI:** 10.1093/geroni/igae098.3802

**Published:** 2024-12-31

**Authors:** Aastha Dharia, Wassim Tarraf, Ramin Homayouni, Mohammad Usama Toseef

**Affiliations:** Oakland University William Beaumont School of Medicine, Rochester, Michigan, United States; Wayne State University, Detroit, Michigan, United States; Oakland University William Beaumont School of Medicine, Rochester, Michigan, United States; Corewell Health, Royal Oak, Michigan, United States

## Abstract

Dual-eligible beneficiaries (enrolled in both Medicare and Medicaid) remain a growing population with complex care needs. However, there are limited studies evaluating self-reported access to healthcare among these individuals. Using six key outcomes that describe the characteristics of beneficiaries’ usual source of care from the 2021 Medical Expenditure Panel Survey, we retrospectively analyzed self-assessment questionnaire data among individuals age 18 and older (N = 12,924) to evaluate the association between healthcare access and dual-eligibility status based on enrollment in managed care. A series of survey-weighted multivariable logistic regression models were estimated. After adjusting the models for demographic factors, socioeconomic factors, and interaction term between dual-eligible and managed care status, we calculated marginal effects of managed care and dual-eligibility on healthcare access outcomes and used analysis of variance contrast tests to estimate the difference in the probability of outcomes based on managed care and dual-eligibility status. The results demonstrate non-dual managed care beneficiaries were significantly less likely to seek care [4.1% versus 2.5%, p-value = 0.0338] and experienced greater delays in care [6.6% versus 4.9%, p-value = 0.0398] due to cost. We also noted no significant impact on established and regular care access independently based on dual-eligibility status or managed care status. These findings suggest managed care may not significantly influence dual-eligibles’ access to healthcare but should be analyzed further for associations with healthcare disparities before making policy recommendations. Future research should also examine associations between quality of care measures and dual-eligibility status to continue holistically evaluating healthcare equity.

